# Investigating the regulation of the *unc-33* promoter by environmental stressors

**DOI:** 10.17912/micropub.biology.000651

**Published:** 2022-10-14

**Authors:** Bianca Garcia-Gonzalez, Sarah Avant, Angelica Carassco-Pena, Maria C Miranda, Kayley Salazar, Eric Torres, Andrea Holgado

**Affiliations:** 1 St. Edward's University

## Abstract

Environmental factors such as prenatal stress are hypothesized to contribute to the development of schizophrenia. Lee and colleagues determined rats exposed to prenatal stress exhibited decreased levels of only one protein, DPYSL2, in their prefrontal cortex and hippocampus. DYPSL2, a protein seen to be inactivated in schizophrenic patients, is important for neuronal development. The *C. elegans* homolog of DPYSL2, UNC-33, is also found to be critical for axonal outgrowth and synapse formation. Herein, we study the effects of environmental stressors such as increasing temperatures and pathogens on the expression of GFP driven by the *unc-33* promoter. Results indicate that neuronal GFP expression was lower in *C. elegans* exposed to these prenatal stressors, making this the first report denoting an environmental regulation of the *unc* -33 promoter. This study provides insight into *unc-33* and the regulation of its expression in relation to temperature and infection.

**Figure 1.**
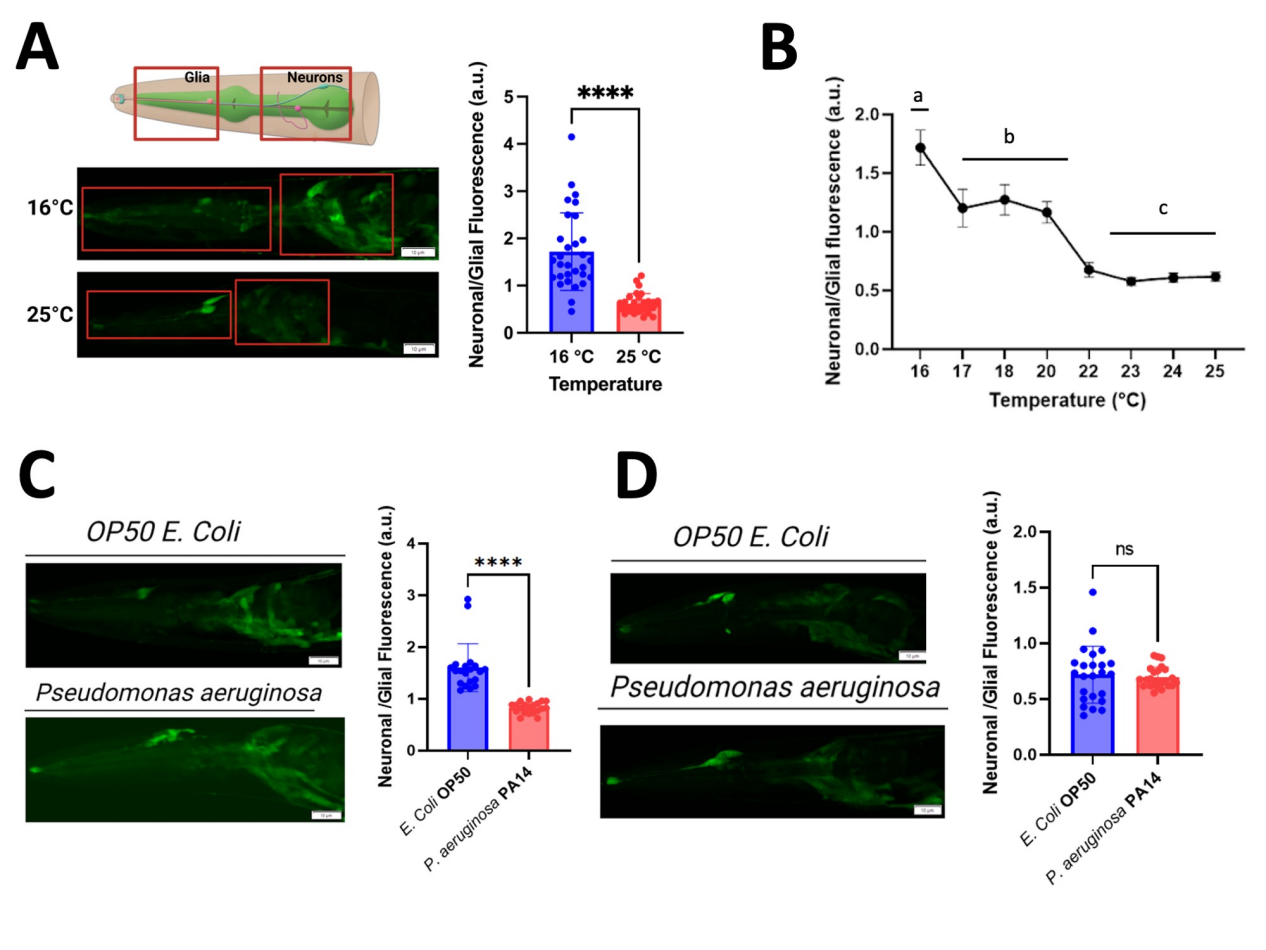
**A** : ***Temperature affects neuronal expression of GFP driven by the unc-33 promoter.*** Representative confocal micrographs of the head region of *C. elegans* nematodes grown on OP50 at either 16°C or 25°C are shown on the left. Quantification of the mean maximum fluorescence plotted as the ratio of fluorescence intensity in neurons over glial cells is shown on the right. Plotted is the mean ± SD, n=30 nematodes/strain/condition. The Mann-Whitney test for non-parametric samples denotes a statistically significant difference with a p-value *<* 0.0001 (****) *.* **B: *The neuronal expression of GFP driven by the unc-33 promoter is significantly changed by Temperature.*** Temperature tolerance curve was constructed by quantifying the ratio of fluorescence intensity in neurons over glial cells of nematodes grown at temperatures of 16°C to 25°C. Plotted is the mean ± SD, n=30 nematodes/strain. One way ANOVA with post hoc Tukey's honestly significant difference (HSD) test denotes statistically significant differences between groups labeled as “a” (16°C), “b” (17-20 °C) and “c” (22-25 °C), p-value *<* 0.01 (**). **C: *Nematodes grown in different bacterial types showed a statistically significant difference in neuronal expression of GFP driven by the unc-33 promoter at 16°C.*** Quantification of the neuronal to glia fluorescence ratio of *C. elegans* incubated on plates seeded with *E.coli* OP50 or *P. aeruginosa* PA14 at 16°C. Plotted is the mean ± SD, n=20 nematodes/strain. The Mann-Whitney test for non-parametric samples denotes a statistically significant difference with a p-value *<* 0.0001 (****) *.* **D: *Nematodes grown in different bacterial types showed no significant difference in neuronal expression of GFP driven by the unc-33 promoter at 25°C.*** Quantification of the neuronal to glia fluorescence ratio of *C. elegans* incubated on plates seeded with *E. coli* OP50 or *P. aeruginosa* PA14 at 25°C. Plotted is the mean ± SD, n=24 nematodes/strain. The Mann-Whitney test for non-parametric samples denotes no significant difference (ns) between the two samples.

## Description

Schizophrenia is a complex and chronic psychiatric disorder that is characterized by positive symptoms, negative symptoms, and cognitive impairment, some of which presents themselves as hallucinations and delusions, lack of emotional responsivity, and difficulty processing information respectively (Farah 2018). The development of schizophrenia is multifactorial in which non-genetic components are involved in conjunction with the effect of genes (Sullivan 2005). While there are no single causal molecular players or environmental factors leading to schizophrenia, prenatal stressors such as nutritional deficiency, maternal stress, and viral-bacterial infections have been shown to increase vulnerability to the disorder (Brown and Patterson 2011; Susser and Lin 1992; Wang and Zhang 2017).

Psychosocial maternal stress during key periods of neurodevelopment leads to the increased risk of activating immune responses that could affect the fetus (Reynolds 2020). During fetal development, nutritional deficiency, maternal stress, and infections have been consistently associated with an increased risk of developing schizophrenia in the fetus (Reynolds 2020). It has been hypothesized that these stressors can cause immune activations, such as the release of cytokines, within the mother, that can alter neuronal development in the fetus (Rakers *et al.* 2020). The release of the inflammatory stress mediator could potentially compromise the blood-brain barrier and cause inflammation leading to neuropsychiatric disorders (Dawidowski *et al.* 2021).

In addition to immune responses, nutritional deficits in mothers cause an inadequate intake of vitamins that cumulatively impact the proper neuronal development through effects on neuronal morphology, receptor affinity, and neurotransmitter production in their offspring (Kwon and Kim 2017). Epidemiological evidence obtained from two individual instances of famine, the Dutch Hunger Winter of 1944-1945 and the Chinese famine 1959-1961, has identified intrauterine exposure to nutritional deficits as one of the contributing factors for the development of schizophrenia (Susser and Lin 1992; Wang and Zhang 2017). Through clinical and sociodemographic data analysis, it was observed that those conceived or in early gestation during the peak of the famine faced a 2-fold risk of developing schizophrenia (Susser and Lin 1992; Wang and Zhang 2017).

Similar to humans, rodent offspring that were a product of maternal gestational stress have shown increased vulnerability to neurodevelopmental changes that may lead to schizophrenia (Lee *et al.* 2015). Lee and colleagues exposed pregnant rats to a series of stressors during a period analogous to a human’s critical period of brain development. Lee and colleagues conducted proteomic and behavioral analyses of prenatally stressed preadolescents and non-stressed controls (Lee *et al.* 2015). Through the evaluation of the prenatally stressed offspring it was revealed that out of an array of proteins isolated from the pre-frontal cortex and hippocampus, two regions often implicated in schizophrenia, the expression of Dihydropyrimidinase-like 2 (DPYSL2) was the only protein suppressed in these regions (Lee *et al.* 2015). In addition to the downregulation of DPYSL-2, schizophrenia-like behaviors were noted in the prenatally stressed offspring (Lee *et al.* 2015).

Converging evidence in regard to DPYSL-2 and the risk of schizophrenia have led researchers to hypothesize that the development of the disorder could be related to decreased amounts of DPYSL2 (Pham *et al.* 2016). DPYSL2 also referred to as Collapsin response mediator protein 2 (CRMP2) is a protein coded by a gene found within chromosome region 8p21, which is involved in axonal elongation and dendritic pruning (Zhao *et al.* 2006). Decreased levels of CRMP2 are associated with a decrease of dendritic spine density on pyramidal neurons which in turn could result in impaired neuronal activity associated with schizophrenia (Moyer *et al.* 2015; Mizutani *et al.* 2019).

Found in neurites, UNC-33, the *C. elegans* homolog of DPYSL2/CRMP2, is essential for proper axonal formation and elongation (Pham *et al.* 2016). UNC-33 works with UNC-44 and UNC-119 to immobilize microtubules in mature neurons (He *et al.* 2020). UNC-33 has three isoforms identified by Tsuboi and colleagues as UNC-33L, UNC-33M, and UNC-33S. For proper UNC-33 function, the dimer formation between UNC-33M and UNC-33S must happen (Tsuboi *et al.* 2005). UNC-33L is highly expressed in the nerve ring, as seen in research by Maniar and colleagues (Maniar *et al.* 2011). The localization of the protein in the nerve ring is hypothesized to be a factor in axonal elongation in *C. elegans* . *unc-33* mutants display defects in axonal outgrowth and alterations to the localization of the UNC-33 proteins (Maniar *et al.* 2011). *unc-33* mutations lead to defects in microtubule elongation, altered microtubule orientation, and significant microtubule sliding, which together produce early termination of axons and mislocalization of axonal proteins to dendrites (Tsuboi *et al.* 2005; He *et al.* 2020)

To expand our understanding of Schizophrenia and investigate the role of environmental stressors regulating the expression of UNC-33/CRMP2/DPYSL2, we examined the activity of the *unc-33* promoter under conditions of increasing temperatures and pathogenic infection. To this end, we used a transgene containing a 2.7 Kb genomic fragment that is immediately upstream to the start site of the *unc-33* gene driving the expression of GFP (Altun-Gultekin *et al.* 2001). Initially, we examined the effects of temperature on synchronous larva 3 (L3) *C. elegans* grown at 16°C or 25°C and quantified GFP fluorescence in neurons and glial cells. Analysis of the fluorescence ratio of neuronal to glial GFP of 30 nematodes per condition shows that at 25°C, neuronal expression of GFP is significantly decreased while the glial expression of GFP is increased. This finding suggests that the activity of the *unc-33* promoter in neurons is regulated by external factors and suppressed upon the exposure to a temperature of 25°C (Figure 1 A). To further investigate the effect of temperature on this promoter, we created a temperature tolerance curve by quantifying the GFP fluorescence in neurons and glial cells at temperatures that ranged from 16°C to 25°C. Quantification of neuronal to glial GFP ratios uncovered three temperature dependent responses. Specifically, neuronal expression of GFP driven by the transgene was significantly different at 16°C, 17-20°C, and 22- 25°C. Nematodes grown at temperatures of 16°C had significantly higher neuronal expression of GFP driven by the *unc-33* promoter compared to the other temperatures analyzed. *C. elegans* grown at 17-20°C had an intermediate ratio of neuronal to glial GFP fluorescent and those incubated at 22- 25°C had the lowest neuronal expression of GFP. Together, the data show that the temperature range of 22°C to 25°C has the highest suppressing effect on the activity of the *unc-33* promoter in neurons. Moreover, results conveyed a negative trend, indicating that as temperature increases the ratio of neuronal to glial fluorescence decreases (Figure 1B).

These findings led us to investigate whether bacterial infection, as a stressor, regulates the activity of the promoter controlling neuronal levels of UNC-33. *Pseudomonas aeruginosa* is a type of gram-negative bacteria that commonly inhabit soil and water. *Pseudomonas aeruginosa* , described as an opportunistic and resilient pathogen, affects humans and animals alike through its biofilm formation, quorum-sensing, and toxin and siderophore secretion (Mahajan-Miklos *et al.* 1999; Kirienko *et al.* 2013). The *daf-16* gene is hypothesized to activate *C. elegans* immunity upon the presence of pathogens such as *Pseudomonas aeruginosa* (Tan *et al.* 1999). Through electron microscopy, it was found that *C. elegans* exposed to *Pseudomonas aeruginosa* for a duration of 24 or 48 hours presented negative effects such as intestinal enlargement and matrix accumulation (Tan *et al.* 1999). Herein we found that after quantifying the ratio of neuronal to glial fluorescence of nematodes cultivated at 16°C in *Pseudomonas aeruginosa* PA14, there was a significantly lower expression of GFP driven by the *unc-33* promoter in neurons (Figure 1C). Conversely, at an incubation temperature of 25°C, nematodes showed no further suppression of neuronal GFP expression suggesting that temperature and *Pseudomonas aeruginosa* PA14 may regulate the activity of the *unc-33* promoter through a similar mechanism (Figure 1D).

Taken together, these findings reflect that adverse environmental conditions, such as high temperatures and pathogen exposure, led to a decrease in neuronal GFP driven by the *unc-33* promoter. These two different stressors seem to have the opposite effect on glial cells as compared with neurons, increasing the activity of the *unc-33* promoter; hence, the heightened level of GFP expressed in those cells. Even though another promoter driving GFP has not been investigated herein, this observation suggests that these stressors are not causing a general effect on transgene expression. Furthermore, it is intriguing to discover that two independent stressors, high temperature and pathogen exposure, have the same effect on the *unc-33* promoter driving the expression of GFP. Future experiments can investigate whether the reduction in promoter activation in neurons is due to epigenetic changes in the region of the genome. Gene expression can be regulated and altered by environmental conditions through epigenetics. Exposure to malnutrition during prenatal development causes epigenetic changes in animal models via DNA methylation (Tobi *et al.* 2014). Additionally, researchers showed that in humans, prenatal stress due to famines led to epigenetic changes linking environmental stressors to an increased risk of developing schizophrenia (Tobi *et al.* 2014). Periconceptional exposure to famine was associated with a 5.2 % reduction in DNA methylation (Heijmans *et al.* 2008). It is warranted to explore alternative avenues by which the activity of the *unc-33* promoter is modulated by external factors and potentially transmitted to subsequent generations via epigenetic changes.

## Methods

*Synchronization of nematodes*

To synchronize nematodes to an L3 stage, adult gravid hermaphrodites from each strain were floated in DI water, transferred to 15mL conical tubes, and centrifuged for 1 minute at 8,000 x g at room temperature. After centrifugation, the supernatant was discarded, and 7 mL of alkaline bleach and 7 mL of water were added. The tube containing the pelleted worms and diluted alkaline bleach was incubated for 7 minutes on a rocker at room temperature. After incubation, the worm suspension was centrifuged for 1 minute at 8,000 x g at room temperature, and the supernatant was discarded. Next, the pellets were washed using 1 mL of M9 buffer and centrifuged for 1 minute at 8,000 x g at room temperature after each wash. A total of three washes were performed. After the final centrifugation, the supernatant was discarded, and the pellets containing eggs were pipetted onto their respective plates seeded with *E. coli* OP50.

*Incubation Conditions to study the effect of temperature*

To analyze the effect of temperature on the activity of the *unc-33* promoter, we incubated nematodes on plates seeded with *E. coli* OP50 at various temperatures within the experimental range of 16°C<X<25°C. To obtain L3 nematodes for imaging, we incubated embryos for 48 hours at 16 °C, 43.5 hours at 17 °C, 39 hours at 18 °C, 30 hours at 20 °C, 27.6 hours at 22 °C, 26.4 hours at 23 °C, and 24 hours at 25 °C prior to imaging.

*Incubation Conditions to study the effect of bacterial infection*

An overnight culture of *Pseudomonas aeruginosa* PA14 was used to seed NGM plates. Seeded plates were left to dry at 37°C for 24 hours. Once dried, synchronized *C. elegans* embryos were plated on seeded plates and incubated at the appropriate temperature until an L3 stage is reached.

*The use of fluorescence microscopy for quantification*

For imaging, eight nematodes were mounted onto a 2% agarose pad with 5µL of 100mM Levamisole. Nematodes were imaged using an Olympus Fluoview Confocal Laser Scanning Microscope (FV3000) and Z-stacks were obtained. To quantify fluorescence, the CellSens software was used to draw regions of interest (ROI) around glial cells and neurons. ROIs were converted to ‘Dynamic ROI over Z’ and the Mean Maximum Intensity Value was plotted as the ratio of Neuronal/Glial fluorescence.

*Statistics*

Statistical analyses were performed using GraphPad Prism version 9.0. The error bars represent standard deviations and statistical differences were plotted as *p < 0.05, **p < 0.01, and ***p < 0.001. Statistical analysis of data from two groups was performed using the Mann-Whitney test and analysis of data from three or more groups was done using One way ANOVA with the post hoc Tukey's multiple comparisons test.

## Reagents

*Strain*

OH438 *unc-4(e120)* II; otIs117 [ *unc-33p* ::GFP + *unc-4* (+)]. IV strain was obtained from *C. elegans* Genetics Center.
